# To understand muscle you must take it apart

**DOI:** 10.3389/fphys.2014.00090

**Published:** 2014-03-11

**Authors:** Christopher Batters, Claudia Veigel, Earl Homsher, James R. Sellers

**Affiliations:** ^1^Department of Cellular Physiology and Centre for Nanosciences (CeNS), Ludwig-Maximilians-Universität MünchenMünchen, Germany; ^2^Physiology Department, University of California Los AngelesLos Angeles, CA, USA; ^3^Laboratory of Molecular Physiology, National Heart, Lung and Blood Institute, National Institutes of HealthBethesda, MD, USA

**Keywords:** muscle, myosin, actomyosin, ATPase, electron microscopy, *in vitro* model

## Abstract

Striated muscle is an elegant system for study at many levels. Much has been learned about the mechanism of contraction from studying the mechanical properties of intact and permeabilized (or skinned) muscle fibers. Structural studies using electron microscopy, X-ray diffraction or spectroscopic probes attached to various contractile proteins were possible because of the highly ordered sarcomeric arrangement of actin and myosin. However, to understand the mechanism of force generation at a molecular level, it is necessary to take the system apart and study the interaction of myosin with actin using *in vitro* assays. This reductionist approach has lead to many fundamental insights into how myosin powers muscle contraction. In addition, nature has provided scientists with an array of muscles with different mechanical properties and with a superfamily of myosin molecules. Taking advantage of this diversity in myosin structure and function has lead to additional insights into common properties of force generation. This review will highlight the development of the major assays and methods that have allowed this combined reductionist and comparative approach to be so fruitful. This review highlights the history of biochemical and biophysical studies of myosin and demonstrates how a broad comparative approach combined with reductionist studies have led to a detailed understanding of how myosin interacts with actin and uses chemical energy to generate force and movement in muscle contraction and motility in general.

## Early studies on muscle fibers

**Figure 1:**
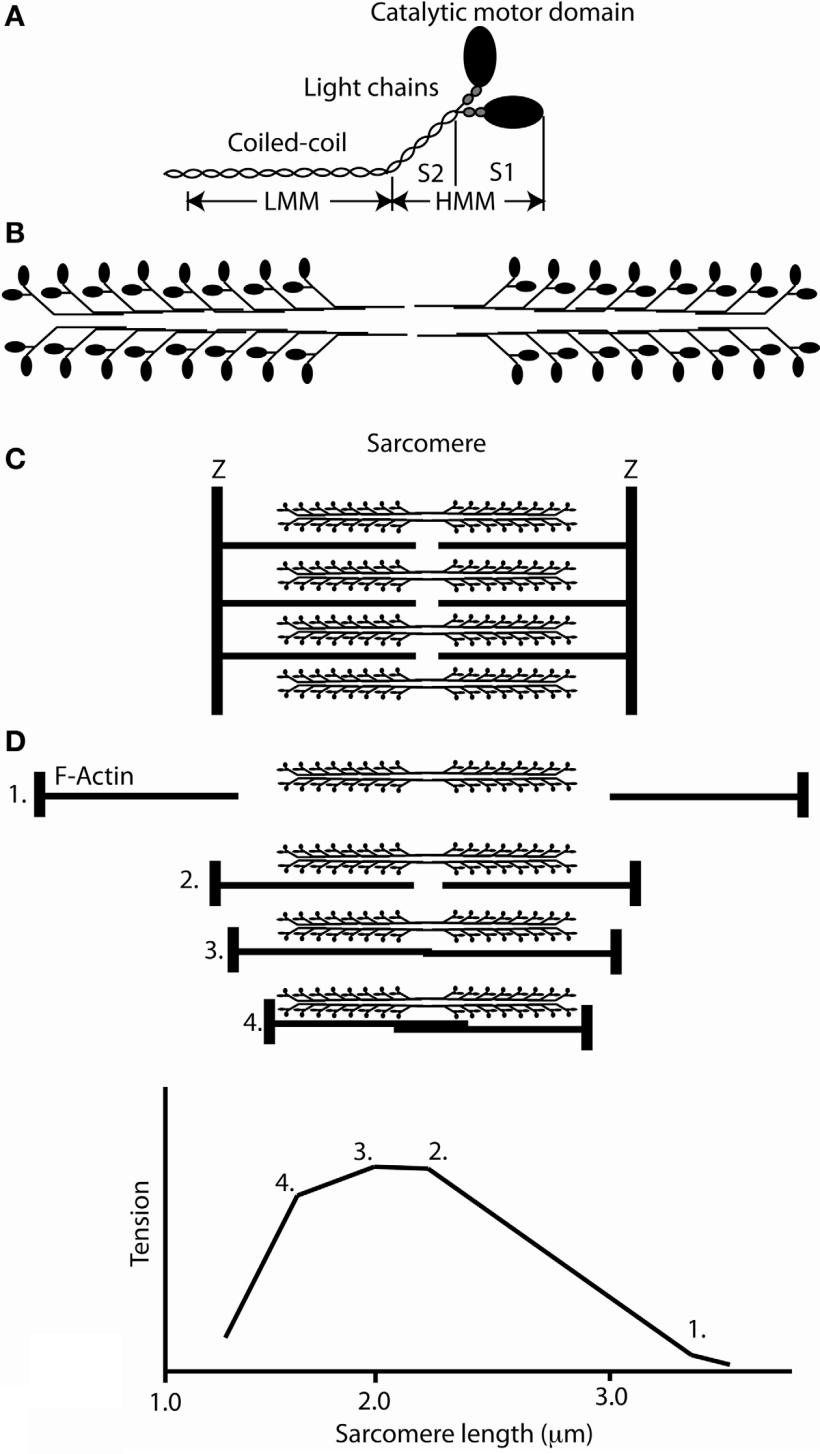
**The structure of myosin and the organization of the sarcomere. (A)** Schematic cartoon of a skeletal muscle myosin molecule showing the molecule to be a hexamer composed of two heavy chains and two pairs of light chains. The heavy chains dimerize to form a coiled coil which forms the elongated tail of the molecule. The major domains that were initially delineated by controlled proteolysis, HMM, LMM, S2, and S1 are marked. **(B)** A schematic of the self-association of myosin molecules to form a bipolar thick filament. **(C)** The organization of actin and myosin in a sarcomere. **(D)** Upper panel, sarcomeres at various length positions; lower panel, the length tension curve corresponding to the sarcomere lengths above.

The mechanism of muscle contraction has been one of the great biological questions and has occupied the attention of many scientists for much of the last half of the 20th century through to today (Huxley, 1996). With its precise order consisting of inter-digitating filaments of actin and myosin, muscles have been amenable to studies using a variety of techniques (Figure 1C). Whole muscles could be connected to various transducers and the muscle could be made to undergo many cycles of contraction and relaxation. It was possible to measure the time course of force development, shortening, lengthening, stiffness, and energy liberation. Fenn and colleagues (Fenn, 1923, 1927; Fenn and Marsh, 1935) showed that the total energy liberated by the muscle was equal to the sum of the heat liberated in an isometric contraction and the work the muscle did when shortening. They later showed there was a hyperbolic relationship between the load against which the muscle shortened and the speed of shortening. The classic paper of Hill (1938) followed in which he showed that there was a hyperbolic relationship between the load against which the muscle shortened and the speed of shortening, and that there was a production of an extra amount of energy released during shortening beyond the work done in lifting the load. In this same era, Myerhof and Lohman (1932) demonstrated that biochemical processes in the muscle were coupled through phosphate transfer and Lipmann (1941) identified ATP as possessing a high energy phosphate bond. In 1940 Ramsay and Street began using single muscle fibers to more precisely measure how muscle force production is related to muscle length (Ramsey and Street, 1940). Similar studies, with even greater time resolution (to the millisecond domain), were extended to single intact and permeabilized muscle fibers (Huxley and Simmons, 1971). With the latter system cytoplasmic solution in the fiber lattice could be replaced by solutions of different ionic composition characteristic of various physiological contractile states and the contractile response measured. The highly ordered array of actin and myosin filaments allowed these model systems to be studied using light and electron microscopy as well as by X-ray diffraction (Huxley and Hanson, 1954; Haselgrove and Huxley, 1973).

A fundamental conceptual advance was presented in two papers published in 1954 which gave rise to the sliding filament hypothesis of muscle (Huxley and Hanson, 1954; Huxley and Niedergerke, 1954). These papers demonstrated that actin and myosin existed as separate polymeric filaments that slid past each other during shortening (Figures 1C,D). The proposal was made that there were individually stochastically active force generation units along the thick filaments that interacted with actin. Using X-ray diffraction studies of the contracting muscle, Huxley (1969) suggested that the cross bridges (individual myosin heads, see Figures 1A,B) exerted a force on the thin filaments by a “lever” action from a rotation of the myosin molecule about its attachment to the thin filament. At the same time Huxley and his collaborators (Gordon et al., 1966) using single fibers and a length clamp arrangement showed that the isometric tension at longer muscle lengths was proportional to the overlap between the thick and thin filaments (Figure 1D) and that the unloaded shortening velocity did not significantly change during shortening from sarcomere lengths from 3.1 to 2.0 μm. These results suggested that independent force generators were uniformly spaced along the thick filaments. Hill and Howarth (1959) showed that during forcible lengthening of muscles during contraction markedly increased the force exerted by the muscle (up to 2 times greater than the isometric force) but, at the same time markedly reduced (in some cases almost to zero) the metabolic energy consumed during the lengthening. These results suggested that the elevated strain on the cross bridge might cause cross bridges to detach before they completed a hydrolysis cycle or reversed the ATP hydrolysis. Finally Huxley, Simmons and coworkers (Huxley and Simmons, 1971; Ford et al., 1977) measured the force and stiffness in single contracting muscle fibers following a sudden rapid change in length. They found that this produced force and stiffness changes which were directly proportional to the amount of filament overlap, and they concluded that the fiber's instantaneous elasticity resided in the cross bridges. These results and conclusions suggested that the displacement produced during a cross bridge attachment-detachment cycle is between 8 and 13 nm and they concluded that the instantaneous elasticity resides in the cross bridges themselves which are the structures responsible for force development. Furthermore, the maximal isometric force exerted by cross bridges in frog muscle fibers was measured at 0.3 N/mm^2^ cross sectional area along with the EM measurements of 500 thick filaments/μm^2^ muscle cross sectional area and about 150 myosin molecules per half sarcomere lead to a forces of about 4 pN per cross bridge. Since only one third to one half of the cross bridges were attached during an isometric tetani, the estimate of force per attached cross bridges about is 8–12 pN (Piazzesi et al., 2002).

However, numerous questions arising from the known structure of muscle and its mechanics were difficult or impossible to answer using only muscle fibers. These include questions such as: What are the molecular arrangements that occur within myosin to move actin? How far does myosin move actin with each power stroke? Is each power stroke accompanied by the hydrolysis of one ATP molecule? What is the reaction mechanism of actomyosin ATP hydrolysis? How much force does a single myosin generate? How does load alter the kinetics of the intermediates in the cross bridge cycle? What is the stiffness of the actin-myosin bond? Do the two heads of myosin bind simultaneously to actin to generate force? What kinetic steps are rate limiting in the myosin ATPase cycle? Which amino acids in myosin are essential for actin binding (and vice versa)? In which ways do the physiological ensembles of myosins affect the kinetics and production of force by the individual force generators?

Historically, the muscles of frog and rabbit were primarily used for physiological and mechanical studies. These tissues were readily available and could be easily isolated from the animals. With the proper supply of metabolic energy in an oxygenated and buffered extracellular solution the contractile behavior of the muscle tissue could be reproducibly studied and maintained in a viable state for several hours. Comparative approaches were used to study the differences between fast and slow muscle fibers from within a single animal and between muscles of different animals (Barany, 1967; Bottinelli et al., 1991). Although evolutionarily distant, it was discovered that insect flight muscle (IFM) exhibits similar biochemical and mechanical characteristics, differing only in certain aspects befitting its function (Reedy et al., 1965; Pringle, 1967; Lehman et al., 1974; Wray, 1979), IFM was shown to possess a very high degree of sarcomere order which made it ideal for electron microscopic and X-ray diffraction studies (Reedy et al., 1965). These studies showed that in rigor muscle, i.e., in the absence of ATP, the myosin cross bridges bound to actin at a 45° angle with respect to the actin filament axis. The comparative studies of various vertebrate and insect muscle were more recently complemented by the creation of transgenic animals, focusing on the effect of myosin mutations in mouse models (Luther et al., 2008).

## *in vitro* experiments defined the structure of the myosin molecule and its interaction with actin

Studies to biochemically define muscle proteins in solution began with the purification of myosin and actin. Albert Szent-Gyorgyi showed that when an actomyosin solution at high ionic strength was exuded into a solution of lower ionic strength, “threads” were produced which contracted upon the addition of ATP (Szent-Györgyi, 1943). Early electron microscope images of rabbit skeletal muscle myosin showed a highly asymmetric structure. Two large polypeptide chains dimerize (Huxley, 1963; Slayter and Lowey, 1967) forming a C-terminal coiled coil tail, while the N-terminus of each chain forms a large globular head (Huxley, 1963; Slayter and Lowey, 1967) (Figure 1A). The bipolar thick filament with a bare zone flanked by regions with projecting myosin heads was formed by polymerization of these myosin dimers (Figure 1B). The initial stage of polymerization creates mini-filaments in which the myosin rods are packed into anti-parallel arrangements and filament elongation occurs by parallel addition of myosins to both sides of this structure (Reisler et al., 1982). Rabbit skeletal muscle myosin can be cleaved into two fragments by trypsin (Figure 1A). The C-terminal light meromyosin (LMM) consisting entirely of the coiled-coil tail retained the solubility properties of the intact molecule (Philpott and Szent-Gyorgyi, 1954). In contrast, heavy meromyosin (HMM) was soluble at low ionic strength and contained two myosin heads each of which retained the actin binding and catalytic site of the molecule (Szent-Gyorgyi, 1953). Electron microscopic studies revealed that HMM contained both heads of the myosin connected by a segment of the coiled-coil that did not self-associate into filaments (Slayter and Lowey, 1967). HMM could be further subdivided into subfragment-one (S1), which contained only a single head of myosin, and subfragment-two (S2), which contained the short section of coiled-coil (Mueller and Perry, 1962; Slayter and Lowey, 1967).

**Figure 2:**
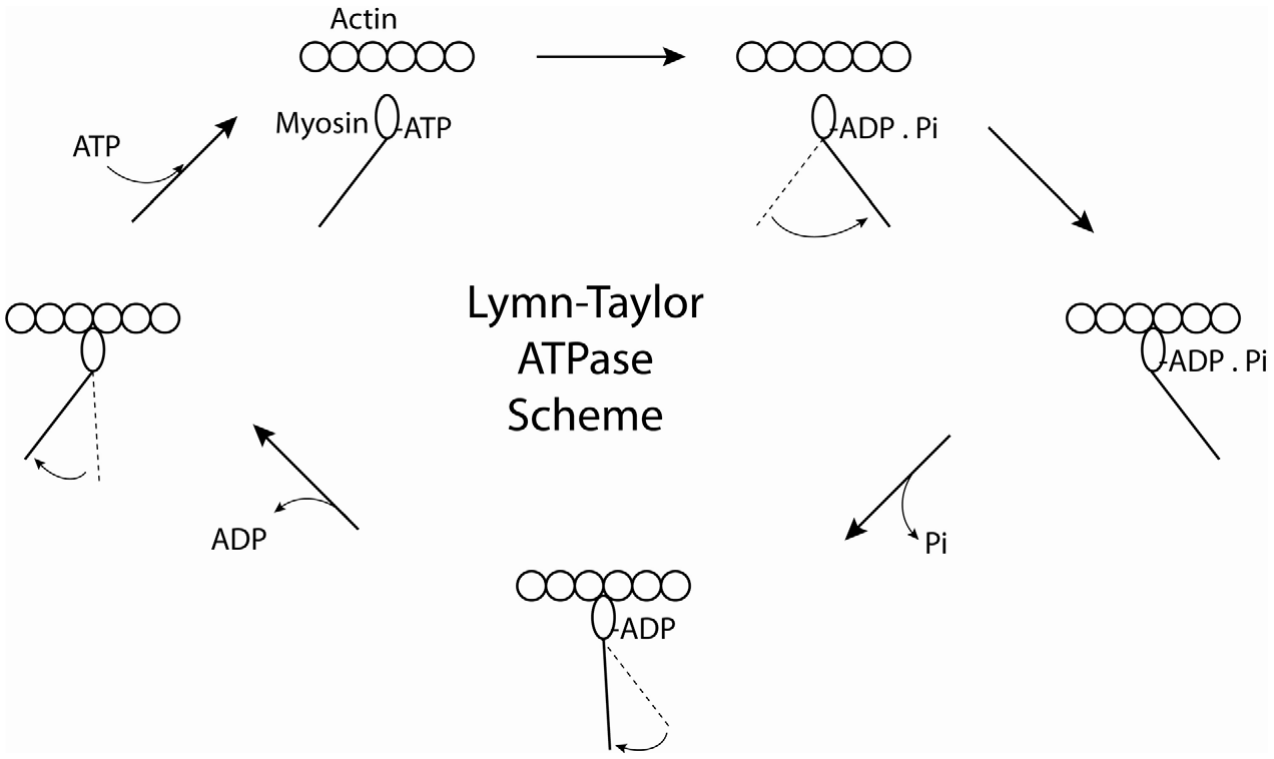
**Cartoon of the Lymn-Taylor Scheme.** The scheme illustrates how the chemical energy obtained from hydrolyzing ATP is converted into mechanical work.

Early studies demonstrated that myosin purified from skeletal muscle was an actin-activated ATPase (Banga, 1942). These initial biochemical studies in solution were greatly hampered by the fact that the interacting myosin and actin filaments tend to aggregate as the ATP is exhausted, a phenomenon known as superprecipitation. The aggregation problem could be avoided by using the dimeric HMM and monomeric S1 fragments, allowing for a detailed study of the acto-myosin ATPase cycle (Bagshaw and Trentham, 1974; Bagshaw et al., 1974). The classical experiments of Lymn and Taylor (1971) demonstrated that S1 alternated between states of high affinity for actin (S1 and S1.ADP) and states of low affinity for actin (S1.ATP and S1.ADP.Pi) during the kinetic cross-bridge cycle and suggested that the myosin cross bridge undergoes a tilting motion to propel actin longitudinally toward the center of the thick filament which is followed by re-priming during the periods of the cycle when myosin is dissociated from actin (Figure 2). The re-priming of the S1 moiety of myosin is associated with ATP hydrolysis. This model provided a kinetic frame work for a cycling cross bridge model for muscle contraction. Transient kinetic studies elucidated the kinetic cycle of skeletal muscle myosin in great detail by the mid 1980's. The rate limiting step was either phosphate release, ATP hydrolysis or some process preceding phosphate release that affects the transition from weak to strong binding states (White et al., 1997). Thus, skeletal muscle myosin spends most of its kinetic cycle in a weakly bound state. The term “duty ratio” was later introduced to quantify the fraction of time a myosin spends in a strongly bound (to actin) state during its kinetic cycle (Uyeda et al., 1990). Skeletal muscle myosin spends only 2–5% of its kinetic cycle in such a strongly bound state and is consequently termed a “low duty” ratio myosin. The use of glycerinated muscle fibers allowed measurements of the kinetics of the ATPase cycle to be studied while monitoring tension, stiffness, and shortening. Caged ATP, caged calcium, caged phosphate, and caged ADP allowed these products to be rapidly released in a muscle fiber by laser flash photolysis. These studies confirmed that rigor muscle was rapidly relaxed by release of ATP and that phosphate could reverse the force-generating tension (Goldman et al., 1982; Dantzig et al., 1992) and see (Goldman, 1987 for review).

Electron microscopy demonstrated that, in the absence of ATP, the S1 region of myosin bound to the actin filament at a 45° angle (Moore et al., 1970) consistent with the earlier EM studies on IFM (Reedy et al., 1965). If the swinging cross bridge model for muscle contraction were correct, one might expect to see myosin heads bound to actin at different angles in the presence of ATP. However virtually no binding was observed in the presence of ATP when the two proteins were mixed by hand and applied to an EM grid. This is consistent with the kinetic studies and the dissociation constant of skeletal muscle S1 for actin in the presence of ATP (Greene and Eisenberg, 1980). The low duty ratio of skeletal muscle myosin has been a major obstacle to studying this acto-myosin interaction *in vitro*. Instead methods were devised for rapidly mixing a preformed actomyosin complex with ATP followed by rapidly staining or cryo-freezing the sample in order to observe the different orientations of the myosin bound to actin in different parts of the ATPase cycle (Frado and Craig, 1992; Walker et al., 1995). In the presence of ATP no discrete binding angle of the myosin head to actin was observed, suggesting a variety of structural conformations. This agreed with studies of the orientation of probes attached to myosin in fibers and with X-ray diffraction (Huxley and Faruqi, 1983; Fajer et al., 1990).

## New approaches lead to new hypotheses

**Figure 3:**
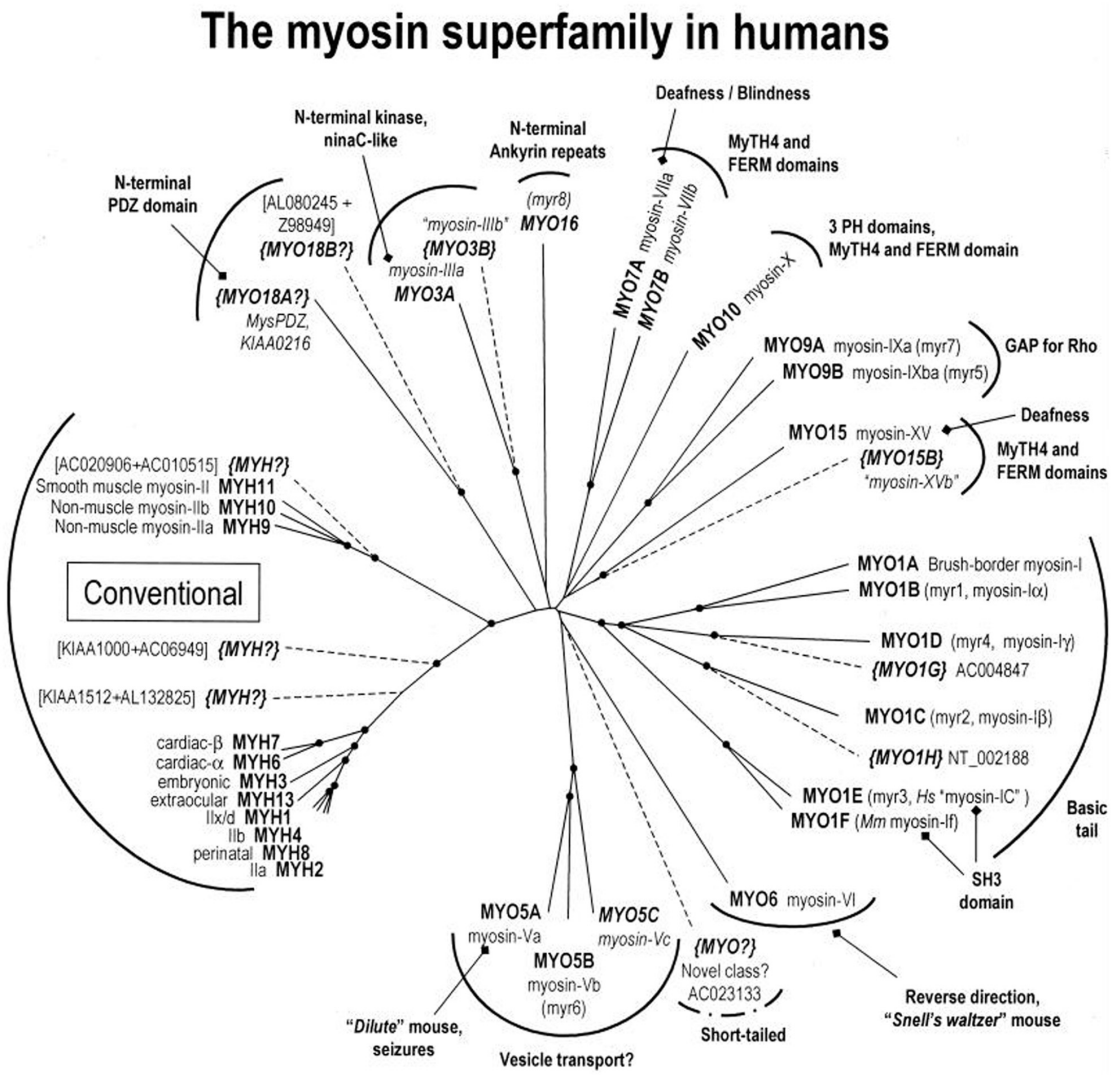
**A myosin phylogenetic tree of the human genome.** Motor domain sequences from all myosins represented in the human genome were analyzed and grouped phylogentically and color coded as to class. Adapted from Berg et al. (2001).

Important technological advances occurred between 1986 and 1994 that greatly accelerated the understanding of how myosin interacts with actin. These include the development of an assay to measure the movement of actin by myosin *in vitro*, the determination of the structure of S1, the ability to create and purify site-directed mutants of myosin and the realization that there was a diverse superfamily of myosins (Figure 3).

**Figure 4:**
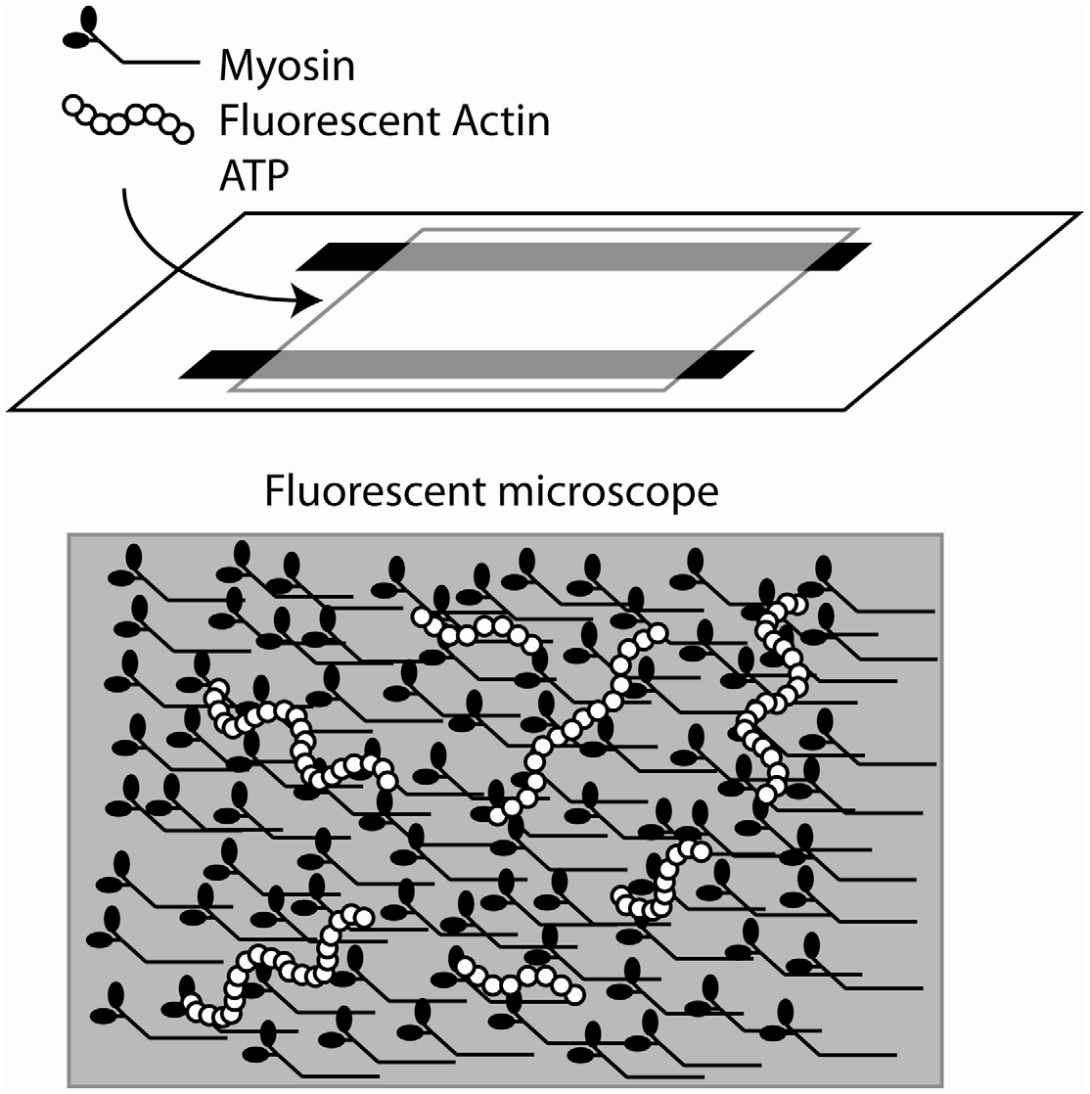
**Cartoon of the sliding actin *in vitro* motility assay. Upper panel**: cartoon of the design of the flow chamber for the assay. A nitrocellulose coverslip is supported by two strips of double sticky tape to create a flow chamber. Myosin is bound to the coverslip and fluorescently-labeled actin filaments are introduced. The reaction is typically started by the introduction of ATP-containing buffer. **Lower panel**: Cartoon of the surface of the coverslip. The bound myosins interact with the actin filament and translocate.

**Figure 5:**
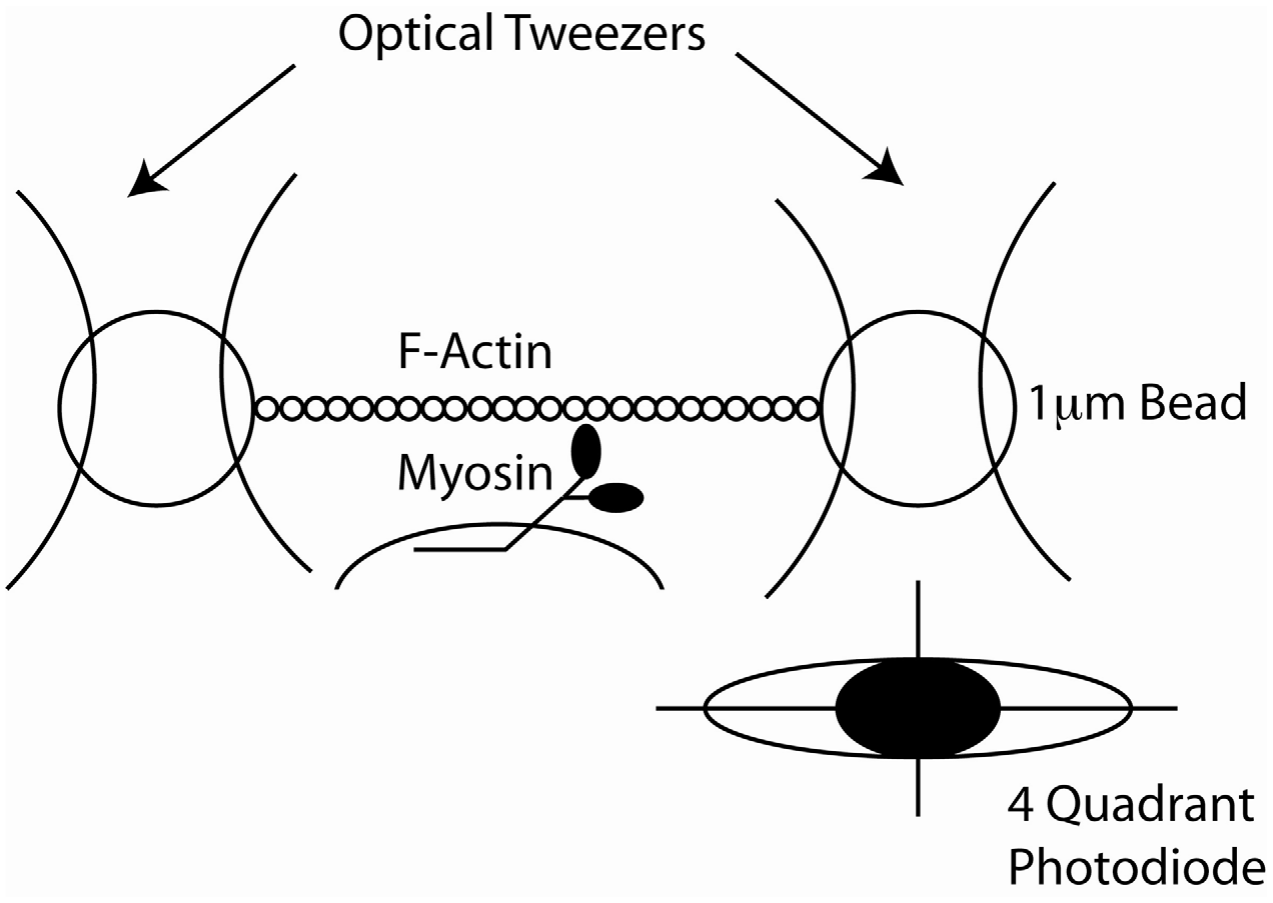
**Cartoon of the design of the three bead optical trap.** Two separate focused laser beams create two optical traps (tweezers). Each trap has a captured bead and there is an actin filament that is bound at each of its end to one of the beads. A single myosin molecule is bound to a bead on the surface of the coverslip. The image of one of the beads is shown to be focused on a quandrant photodiode which detects its position with nanometer accuracy.

Yanagida et al. observed single rhodamine-phalloidin labeled actin filaments, polymerized *in vitro*, using fluorescence microscopy (Yanagida et al., 1984). This observation was used by Kron and Spudich to design an *in vitro* acto-myosin gliding filament assay (Kron and Spudich, 1986), where myosin molecules, from skeletal muscle or the amoeba, *Dictyostelium discoideum,* were immobilized on a glass coverslip surface and moved fluorescently-labeled actin filaments (Figure 4). Subsequent studies showed that the myosin fragment S1 was sufficient to move actin filaments, albeit at reduced speeds (Toyoshima et al., 1987). This assay gave strong support to the widely accepted sliding filament model of muscle contraction and made alternative models less likely (Harrington, 1971; Oplatka, 1989). It also allowed for an estimation of the myosin duty ratio and of the size of the myosin working stroke (Harada et al., 1990; Uyeda et al., 1990, 1991; Harris and Warshaw, 1993). The *in vitro* motility assays led to the development of single molecule mechanical techniques to measure the force and movement produced by a single myosin motor. In one such assay an actin filament was attached to a glass micro-needle. The actin filament was allowed to interact with myosin molecules bound to the surface. The bending of the needle was monitored to measure displacement and force (Kishino and Yanagida, 1988). Various optical tweezers apparatus were designed to measure the interaction of single myosin molecules with single actin filaments (Finer et al., 1994; Molloy et al., 1995a; Ishijima et al., 1998; Rief et al., 2000; Veigel and Schmidt, 2011). In particular the three bead optical trap assay, in which a single actin filament is suspended between two optically trapped plastic beads has been instrumental in understanding myosin's molecular mechanisms (Figure 5). The tethered actin filament is allowed to interact with a single motor molecule immobilized on a pedestal attached to the surface of the experimental chamber. The positions of each of the two beads are monitored by split photodiodes recording sub nanometer displacements on a sub-millisecond time scale (Simmons et al., 1993). Analysis of the change in position of the trapped beads during interactions of the myosin motor with the suspended actin filament can be detected and measured (Batters and Veigel, 2011). These experiments showed that skeletal muscle myosin interacted transiently with actin filaments and that the actin filaments were typically displaced by 5–10 nm with each interaction (Molloy et al., 1995b; Guilford et al., 1997; Mehta et al., 1997; Veigel et al., 1998). This number agreed remarkably well with estimates of the powerstroke made in muscle fibers (Huxley and Simmons, 1971; Ford et al., 1977; Piazzesi and Lombardi, 1995). At low ATP concentrations the lifetime of the interactions were limited by the ATP concentration (i.e., the rate at which ATP binds to and dissociates the actomyosin linkage). Furthermore, using an optical trap with force feedback Takagi et al. (2006) determined that the force per isometric cross bridge is 9 pN which agrees well with estimates from muscle fibers (Piazzesi et al., 2002).

**Figure 6:**
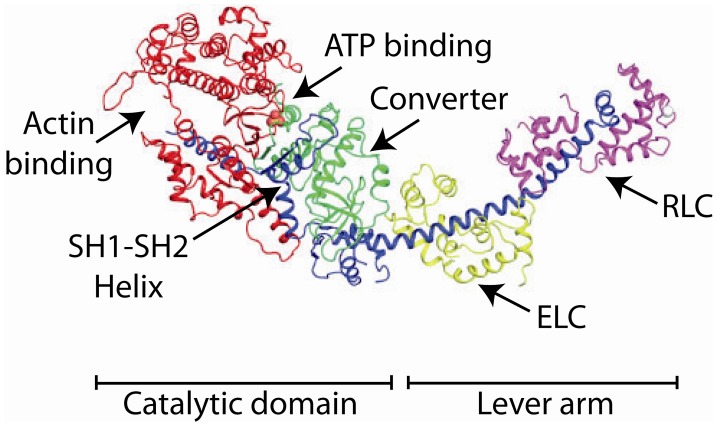
**The crystal structure of chicken skeletal muscle S1.** The heavy chain is colored (from N-terminal to C-terminal sequence) in green, red, and blue. The ELC is colored lime and the RLC is colored magenta. The positions of various domains are marked.

The crystal structure of chicken skeletal muscle myosin S1 containing the motor domain and both the essential (ELC) and regulatory (RLC) light chain in 1993 (Rayment et al., 1993a,b) suggested a molecular explanation for the tilting cross bridge model for muscle mechanics (Figure 6). It revealed that most of the N-terminal portion of myosin S1 was composed of two large domains (historically termed the upper and lower 50 kDA domains) separated by a narrow cleft, but that the C-terminal part of the S1 heavy chain was a single α-helix that was stabilized by the associated essential (ELC) and regulatory (RLC) light chains. It was suggested by Rayment et al. that the stabilized α-helical light chain binding domain forms a mechanical lever arm that might swing about a pivot point to move actin. The crystal structure of the myosin S1 was modeled into a three-dimensional reconstruction of the electron micrographic image of myosin bound to actin in rigor (Rayment et al., 1993a). The crystal structure fit relatively well into the density expected for S1 and gave an indication of what the actin-myosin interface might look like. Several surface loops were identified that were predicted to interact with actin (i.e., as binding sites). Major refinements in electron microscopy methods and instrumentation now allow the structure of the actomyosin complex to be resolved to 8 Angstrom resolution (Behrmann et al., 2012).

To use the information from the crystal structure a system needed to be developed to create mutants. The discovery in 1987 that the amoeba *Dictyostelium* underwent homologous recombination at a high frequency introduced a powerful tool for studying myosins, the ability to create site directed mutants (De Lozanne and Spudich, 1987). Some years later it became possible to produce myosins using the baculovirus/Sf9 system (Trybus, 1994). Scientists have also created transgenic mice bearing mutations in contractile proteins that give rise to various cardiomyopathies (Geisterfer-Lowrance et al., 1996). *Dictyostelium* has a number of advantages over mice when creating mutant myosins. Its haploid genome containing only a single myosin II gene is easily targeted for knock out or replacement by a mutant version of the gene. It can be grown on plates or in suspension cultures from which large quantities of cells can be harvested (Manstein et al., 1991). When grown in suspension culture the myosin knockout cells undergo karyokinesis, but can't complete cytokinesis which results in cells containing multiple nuclei which eventually die. However, the myosin II knockout is not lethal when the cells are grown on a surface since here they divide by a process termed *traction mediated cytofission* (Spudich, 1989). Thus the wild type *Dictyostelium* myosin can be replaced by mutant myosins and the ability of the mutation to rescue cytokinesis was used as a screening tool (Ruppel and Spudich, 1996). In suspension the cells can be grown cheaply in large quantity to provide ample protein for studies and soluble S1 and HMM-like fragments could be produced in large quantities (Manstein et al., 1991). This became the first system for generating mutant myosins either by site directed targeting of conversed residues or by screening for functional mutants in randomly mutagenized cells.

A major benefit of studying *Dictyostelium* myosin was that, unlike chicken skeletal muscle S1, which yielded crystals only after great effort and only with no nucleotide bound, the *Dictyostelium* motor fragments were easily crystallized in a variety of bound nucleotide states and the lever arm was found in different, nucleotide-dependent orientations (Smith and Rayment, 1995). These different conformations were interpreted as corresponding to different states of the myosin in the contractile cycle (Holmes and Geeves, 2000). Crystal structures of smooth muscle myosin S1 (Dominguez et al., 1998) and scallop muscle myosin S1 (both prepared from tissue purified myosin) and its regulatory domain (Houdusse and Cohen, 1996; Houdusse et al., 2000) also contributed significant insight into myosin's structure and function.

The *Dictyostelium* expression system, however, was only useful for making *Dictyostelium* myosin. In the mid 1990's several groups began using the baculovirus *in vitro* expression system to infect an insect cell line (Sf9 cells) to drive the expression of wild type and mutant myosins. The ability of these cells to express mammalian myosins was variable. Several myosins such as smooth muscle myosin II (Trybus, 1994), non-muscle myosin II (Pato et al., 1996), myosin V (Trybus et al., 1999; Wang et al., 2000), myosin VI (De La Cruz et al., 2001), Myosin IX (Nalavadi et al., 2005), Myosin XVIII (Guzik-Lendrum et al., 2013), and Myosin XXI (Batters et al., 2014) expressed in this system. Unfortunately skeletal and cardiac muscle myosins were not expressed in this system, probably because the insect cells lacked the appropriate chaperones (Sweeney et al., 1994; Sata and Ikebe, 1996).

Comparative sequence alignments and the crystal structures of the myosins revealed a number of highly conserved segments. Researchers targeted many of the conserved amino acids for site directed mutagenesis and expressed the mutant constructs in either the *Dictyostelium* or the baculovirus/Sf9 cell expression system. Much of the critical work was carried out on *Dictyostelium* myosin II or smooth muscle myosin. In these myosins the amino acids necessary for catalysis and force generation were quickly discovered. Many of these residues lay in three conserved regions of the myosin sequence, which bears homology to that of G-proteins. The conserved regions are termed switch 1, switch 2, and the P-loop. Their importance is evident when they are mapped onto the crystal structure of myosin with nucleotide bound in which several specific amino acids are involved in nucleotide binding or positioning of a water molecule to carry out a nucleophilic attack on the nucleotide's γ-phosphate (Fisher et al., 1995). There is a conserved arginine (R228, *Dictyostelium* numbering) residue in switch 1 that forms a hydrogen bond with a glutamic acid residue (E459) in switch 2 to close the nucleotide binding pocket to permit catalysis. Mutation of either of these residues to an alanine greatly inhibits the actin-activated ATPase activity and abolishes the ability of the myosin to move actin filaments (Onishi et al., 1997; Furch et al., 1998). These two residues are almost invariantly conserved in all classes of myosins. Interestingly one myosin, myosin 18, has had evolutionary substitutions of its active site that prevent formation of this salt bridge. Thus myosin-18 does not exhibit any measureable actin-activated ATPase activity (Guzik-Lendrum et al., 2013). The pivot point for the movement of the lever arm was shown to be at a glycine residue in a region that was termed the SH1/SH2 helix in skeletal muscle because of the presence of two highly reactive cysteine residues. The location of this helix is shown in Figure 6. Mutation of this glycine residue (G690 in *Dictyostelium*) to even an alanine (replaced of R = H vs. R = CH_3_) uncouples actin-activated ATP hydrolysis from motility (Patterson et al., 1997). Two surface loops, termed loop 1 and loop 2, were identified which have varying sequences amongst the different myosins (Rayment et al., 1993b). Loop 1 lies near the nucleotide binding site and loop 2 at the tip of the motor where it is thought to constitute the initial weak binding interaction with actin. Mutagenesis of these loops revealed their roles in actin binding and determining the kinetics of the molecules (Spudich, 1994). In particular, increasing the positive charge in loop 2 increases the actin affinity in the presence of ATP (Furch et al., 1998). Other regions important for actin binding were identified (Kojima et al., 2001; Onishi et al., 2006; Varkuti et al., 2012).

A major problem encountered in studying vertebrate skeletal muscle myosin either in biochemical experiments or in the context of the muscle fiber has been the lack of ideal labeling positions for spectroscopic probes (See Sellers, 1999). There are two rapidly reacting cysteine residues (C707 and C697, both rabbit skeletal muscle sequence) termed SH1 and SH2 that lie on a helical region just before the converter domain (Rayment et al., 1993b). Much work was carried out using myosin labeled on these sites (Thomas, 1987). However, myosin labeled in this way was subsequently shown to be incapable of translocating actin filaments in the *in vitro* motility assay which rendered the interpretation of the results of experiments using this method of labeling myosin uncertain (Root and Reisler, 1992). One study exchanged an RLC labeled with a bifunctional rhodamine into skeletal muscle fibers (Corrie et al., 1999). The orientation of this dye could be ascertained from the crystal structure of this region. Using the fluorescence polarization of the dye, the motions of the force-generating myosin heads could be studied. The ability to genetically modify and express *Dictyostelium* myosin fragments afforded a means to circumvent this problem. A “cys-lite” *Dictyostelium* motor domain was engineered that allowed researchers to position labeling sites at various locations on the molecule which has proven advantageous in exploring myosin function (Shih et al., 2000). Similar experimental approaches were applied to tryptophan residues in *Dictyostelium* myosin which allowed researchers to elucidate the kinetics of the movements of the switch elements upon binding and hydrolysis of ATP (Malnasi-Csizmadia et al., 2000). These studies provided strong support for the ATP-dependent movement of the lever arm and for the movement of important regions of myosin during an ATPase cycle. Another system for studying myosin function in the context of the muscle was the flight muscle of *Drosophila*. This muscle, while essential for flight, is not essential for viability of the animal. Single muscle fibers could be isolated and studied mechanically and, importantly, mutant myosins could be expressed in this system in place of the endogenous myosin. *Drosophila* has only a single gene for skeletal muscle myosin, but it uses alternatively spliced versions of this gene to populate a variety of muscle fibers with different mechanical properties. Using this system, Bernstein and colleagues have probed the function of critical regions of myosin such as the S2 region, the converter region, and the nucleotide binding regions (Swank et al., 2006; Suggs et al., 2007; Miller et al., 2009; Kronert et al., 2012).

Mutagenesis studies were not possible with skeletal or cardiac muscle myosins during the time period of these important studies since these myosins do not express well in the baculovirus/Sf9 system. However, it was known that mutations in the human β-cardiac muscles myosin were linked to hypertrophic cardiomyopathy (Geisterfer-Lowrance et al., 1996). It was possible to carry out *in vitro* motility experiments and optical trapping studies with biopsy samples from patients with various mutations, but while these studies may have provided insight into the disease mechanism, they did not provide fundamental insight into myosin's function for several reasons (Cuda et al., 1993; Palmiter et al., 2000). Firstly, the experiments were challenging since the patients were heterozygous for the mutation and only small amounts of tissue could be obtained. Intriguingly the mutations had only subtle effects on the measured parameters which might be due to the fact that these patients typically survive into early to late adulthood and some exhibit only subtle symptoms of the disease. The same myosin isoform is also expressed in human slow muscle and the patients manifest little or no impairment of their skeletal muscle contraction. Thus it is likely that a mutation to any amino acids critical to myosin function would not support life and this reinforces the use of model systems where myosin is not essential to the viability of the cells. As more patients have been examined it has become clear that hypertrophic cardiomyopathy can be caused by mutations in many sarcomeric proteins and that mutations of over 200 different amino acids in myosin alone are associated with the disease (Walsh et al., 2010). It should be noted that recently striated muscle myosin has been expressed in skeletal muscle C2C12 cells for biochemical studies (Wang et al., 2003) and that β-cardiac myosin subfragments have been expressed in this system in sufficient quantities to study the molecular properties of HCM mutations (Deacon et al., 2012; Sommese et al., 2013).

**Figure 7:**
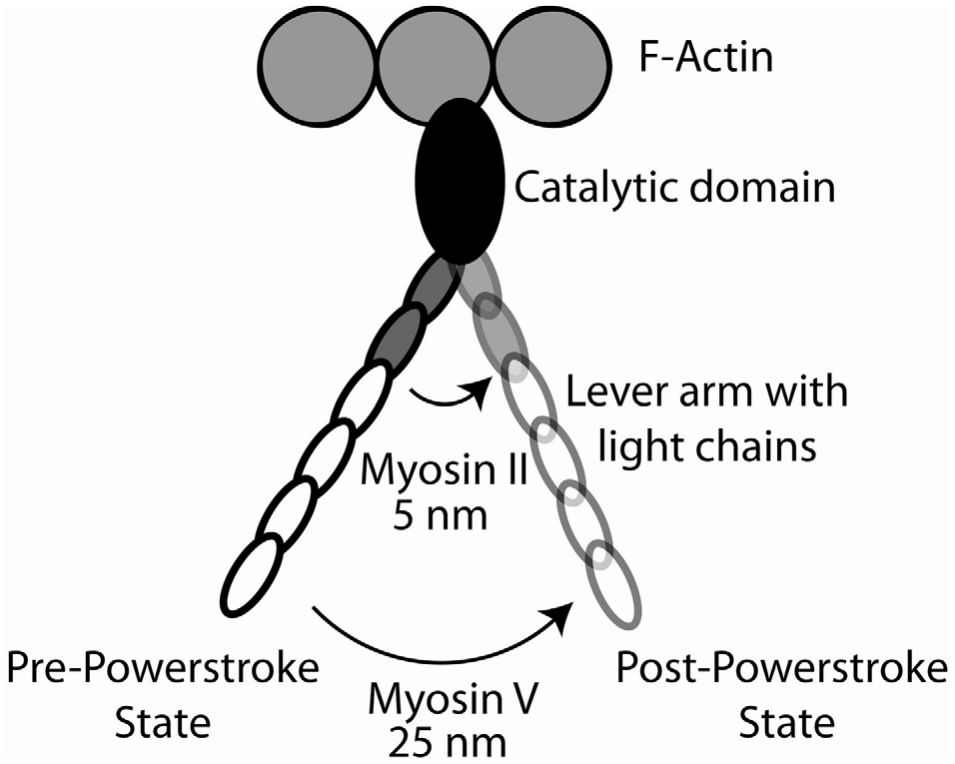
**Cartoon of the myosin cross-bridge powerstroke.** The effect of lever arm length on the effective powerstroke is shown for myosin II (two bound light chains) and myosin V (six bound light chains). The position of the lever arms in the pre-powerstroke and post-powerstroke positions are shown.

In the 1990's it became clear that there was a large superfamily of myosin molecules of which the classical skeletal and cardiac muscle myosins (now termed myosin II) was but one member (Mooseker and Cheney, 1995) (Figure 3). The first “unconventional” myosin had been discovered in *Acanthamoeba castellani*, a soil amoeba, in 1973 by Pollard and Korn (1973) and a strange myosin-like protein containing a kinase domain on its N-terminus (initially termed *ninaC*, but now termed myosin III) was discovered by Montell and Rubin (1988) in *Drosophila* as a gene involved in fly vision. Both proteins had lower molecular weights than skeletal muscle myosin and electron micrographs of the *Acanthamoeba* protein (termed myosin I) showed it to be single-headed without a long tail (Pollard and Korn, 1973). We now know that there are more than 35 classes of myosin as determined by phylogenetic analysis of the motor domains and that humans have 39 myosin genes representing 12 of these classes (Odronitz and Kollmar, 2007). Myosins are found in virtually every organism and there are many myosins expressed in most mammalian cells. The myosins found in cardiac and skeletal muscle are termed class II or “conventional” myosins, although there are also class II myosins found in smooth muscle and non-muscle cells. Myosins have roles in numerous cellular tasks including cytokinesis, trafficking of membranous vesicles within cells, endocytosis, exocytosis, phagocytosis, maintenance of cortical membrane tension, cell adhesion, cell motility, filopodia formation, stereocilia formation and maintenance, and cell signaling (See various chapters in Myosins: A Superfamily of Molecular Motors, Coluccio, 2008). Myosins can be defined as having three major structural divisions, the motor domain, the lever arm and the tail (See Figures 1A, 6). In most classes of myosin the motor domain remains relatively conserved in terms of structural motifs and ability to interact with actin in an ATP-dependent manner. The lever arm of myosin II follows a small domain formed by the heavy chain alone, termed the converter domain and has two binding sites for light chains (IQ motifs), which always bind an ELC and an RLC. The lever arm of unconventional myosins may contain 0–6 IQ motifs and often binds calmodulin instead of or in addition to ELCs and RLCs. The length of the neck is a major determinant of the size of the myosin powerstroke (Figure 7). In addition, the lever arm may contain a stable α-helical (SAH) domain which serve to extend the lever arm length without binding additional light chains (Knight et al., 2005). The tail is the most diverse domain of myosins (Krendel and Mooseker, 2005). The tail determines the oligomerization state of the molecule and in which way the motor molecule is attached to cargo. Various domains are present in the tails. Some myosins have coiled-coil domains which dimerize the molecules, but many do not and are probably monomeric. Other functional domains found in tails include PH (plectstrin homology) domains that mediate binding to membranes, MyTH4-FERM (myosin tail homology-4-band 4.1-ezrin-radixin-moesin) domains and SH3 (src-homology-3) domains that mediate protein-protein interaction, PEST (proline-glutamic acid-serine-threonine) domains that are the site of proteolysis and GAP (GTPase activating protein) domains that are involved in signal transduction pathways. The crystal structures of the myosin motor domains from classes I, V, and VI have been solved and there is remarkable conservation of the basic structure when compared to the structures of myosin II class molecules (Kollmar et al., 2002; Coureux et al., 2003; Menetrey et al., 2005). This makes it likely that insights gleaned from studying these structures are relevant to myosin II. Myosin V was crystallized in several conformations including one that is thought to be a model for myosin bound to actin at the end of the power stroke (Coureux et al., 2004). Myosin VI has a novel insert just after the converter domain which reorients the lever arm and is responsible for this myosin's ability to move in the opposite direction on actin filaments compared to other myosins (Menetrey et al., 2005).

Cryo-electron microscopy (Cryo-EM) of actomyosin complexes has provided a link between the crystal structures and the geometry of myosin bound to actin (Rayment et al., 1993a). Cryo-EM allowed researchers to see the large scale conformational changes linked with the different nucleotide states. This change in conformation associated with ADP and rigor was first seen with smooth muscle myosin-II (Whittaker et al., 1995) and subsequently with myosin-I, myosin-VI, and myosin-V (Jontes et al., 1995; Wells et al., 1999; Volkmann et al., 2005).

A working stroke in two sub-steps was first seen in single molecule optical tweezer experiments with unconventional myosin I (Veigel et al., 1999), and has since been shown to be a common feature of all myosins investigated to date including skeletal muscle myosin-II (Capitanio et al., 2006). This 2-step feature has been shown to be an important feature for force sensing and for the coordination of heads in processive dimeric motors (Veigel et al., 2005).

Many of the biochemical properties that we have described for skeletal muscle myosin II so far make it difficult to study its interactions with actin at a biochemical and electron microscopic level. In the presence of ATP a myosin II head spends most of its kinetic cycle detached from and interacting only weakly with actin so that the lifetime of its strongly bound states are short. However some of the unconventional myosins serve as cargo transporters and have evolved to spend most of their kinetic cycle bound strongly to actin. An example is myosin V which, in melanocytes serves to aid in the transport of pigment granules, termed melanosomes, to the tips of the dendritic processes (for review, see Hammer and Sellers, 2012). In Purkinje neurons myosin V transports endoplasmic reticulum to the tips of the dendritic spines (Wagner et al., 2011). Myosin V is present in most animal genomes. It has a conserved motor domain, a lever arm containing six IQ motifs that bind either all calmodulin or a mixture of calmodulin and essential light chains depending on the species and a tail containing a long segment of coiled-coil which serves to dimerize the molecule and ends in a cargo binding globular tail region. Dimerized Myosin V molecules do not form higher order polymer structures. Biochemical studies showed that myosin V has a high duty ratio meaning that it spends the majority of its kinetic cycle strongly bound to actin (De La Cruz et al., 1999). It accomplishes this by essentially changing two rate constants compared to skeletal muscle myosin. The rate of phosphate release by myosin V is fast and ADP release from the actin-myosin complex is rate limiting (see Figure 2). These kinetics allow single molecules of myosin V to processively move along actin filaments *in vitro*. Despite this kinetic difference both myosin V and myosin II molecules cycle through weakly and strongly attached actin states during their cycle which helps validate the use of myosin V as a model to understand the function of myosin II.

These biochemical properties make myosin V much more amenable to study biochemically, particularly at the single molecule level, than most other myosins and it can be argued that it is the best understood myosin in terms of its mechano-chemical properties (Sellers and Veigel, 2006). Optical trapping studies of myosin V demonstrated that it takes multiple “steps” along actin during a single encounter and that these steps are separated by 36 nm (Mehta et al., 1999; Rief et al., 2000; Veigel et al., 2001). When only single interactions occur (i.e., a single power stroke followed by dissociation) a power stroke size of about 25 nm is detected (Veigel et al., 2001). This is much larger than that seen for myosin II, but is consistent with the neck region acting as a rigid lever arm given that the neck region of myosin V has six bound light chains compared to two for myosin II. The stepping kinetics during a processive run at high ATP is consistent with ADP release being rate limiting for myosin V. The difference between the size of the working stroke of a single myosin V head (25 nm) and the step size of the dimeric motor (36 nm) in these experiments led to the current models as to how the two heads of the processively moving myosin V are coordinated.

The processivity of myosin V can also be observed in a variant of the sliding actin motility assay where one inverts the geometry of the proteins used in myosin II sliding filament assays. In the single molecule myosin V *in vitro* motility assay, actin filaments are bound to the coverslip surface and fluorescently labeled myosins are in the solution. When a labeled myosin V molecule contacts the actin filament a processive run ensues that can be followed to determine the run length and velocity (Sakamoto et al., 2000). If sufficient photons can be captured the instantaneous position of the myosin can be determined within a few nanometers by fitting the point spread function to a two-dimensional Gaussian distribution (Yildiz et al., 2003). This technique confirmed the optical trapping observation that myosin V takes a center of mass movement of 36 nm for each step, however, in this assay the movement of a single myosin motor could be detected as a 72 nm “stride” demonstrating that the two heads moved in a “hand-over-hand” mechanism where the two motor domains alternate leading and trailing positions. This was confirmed in a study where quantum dots of different colors attached to the two heads of a single myosin V molecule could be seen to alternate positions while the molecule processively moved along an actin filament (Warshaw et al., 2005).

**Figure 8:**
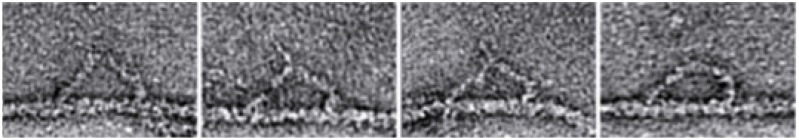
**Myosin V molecules trapped in the actin of moving along actin.** Negatively stained electron micrograph of myosin V bound to actin in the presence of ATP. In each panel, the two heads of myosin are seen to bind to actin separated by 36 nm. Image is taken from Walker et al. (2000).

Electron microscopy of myosin V molecules trapped while moving along actin demonstrated that the molecule bound to actin via both heads and that the two heads were separated by 36 nm (Walker et al., 2000; Burgess et al., 2002) consistent with the step size of the dimeric molecule observed in single molecule mechanical experiments described above (Figure 8). This separation is important to cells since it matches the helical repeat distance of the actin molecule. Binding with this separation allows the molecule to essentially walk “straight” along an actin filament and it need not tightly spiral around the actin filament which would complicate the movement of large membrane cargoes. Importantly, the two heads of myosin were bound in different chemo-mechanical states and through single particle image processing features of the attached molecule could be discerned. The motor domain structure of both the leading and trailing heads were reasonably similar, and the differences were in the position of the converter domain and the lever arm. The lever arm of the trailing head was bound to actin at a 45° angle which is consistent with the post power stroke confirmation of the molecule, but that of the leading head was bound at an angle of about 115° which would be consistent with a myosin head in a pre-power stroke configuration. In particular the position of the converter region could be discerned and in most of the leading heads the converter region was still in the pre-power stroke orientation. Many molecules were attached by only a single head and while some were attached at the post power stroke angle, others were attached at various angles consistent with molecules that were at the start of their power strokes.

Bifunctional attachment of fluorescent probes to a single calmodulin moiety in the lever arm of myosin V allows for detection of the lever arm angular position during processive movement (Forkey et al., 2003). This study showed that the lever arms of the leading and trailing heads assumed different angles as expected from the above static EM pictures. Together these studies provided very strong support of the swinging cross bridge model.

Several mutagenesis experiments support the idea that the light chain binding neck region acted as a lever arm. In these studies the neck regions of smooth muscle myosin II and myosin V were engineered to be longer or shorter than wild type via manipulation of the number of IQ motifs (Warshaw et al., 2000; Sakamoto et al., 2003, 2005). With both myosins the size of the power stroke of a single myosin head and the step size of the processively moving dimeric myosin V molecules were proportional to the length of the lever arm. Another experiment expressed a *Dictyostelium* myosin mutant in which the native lever arm was entirely replaced with an artificial one made up of a rigid section of sequence from α-actinin (Ruff et al., 2001) In the optical trap this mutant gave power stroke sizes that were in proportion to the predicted length of the artificial lever arm.

The question of chemo-mechanical coupling is a crucial one in muscle research. Although most researchers would agree that one ATP molecule is hydrolyzed per power stroke and that load affects the kinetics of the cycle, the details of the chemo-mechanical energy conversion are not fully characterized. Here again, a reductionist approach, often using myosins other than muscle myosin have provided critical information. These experiments revealed that the sensitivity to load is very different in different parts of the cross bridge cycle. Studies of the load dependence of the chemo-mechanical cycle have been performed on striated muscle and smooth muscle myosin II (Veigel et al., 2003; Takagi et al., 2006), myosin class I (Laakso et al., 2008), and myosin class V (Veigel et al., 2005). To date the most detailed studies have been carried out on myosin V. Experiments on dimeric myosin V showed that at a certain load (stall force) the motor will change directionality (Gebhardt et al., 2006) and that processive backward movement along actin is independent of ATP. Here, the energy required for myosin detachment from actin was provided by the applied load. Studies on single myosin V heads on the other hand made it possible to study the load dependence of the different transitions within the chemo-mechanical cycle in detail. They revealed that the most sensitive transition in the cycle was associated with ADP release (Veigel et al., 2005). This provided a molecular explanation for the Fenn effect described for muscle in the 1920's (Fenn, 1923). This effect shows that muscle is able to adjust its energy consumption, and thus the kinetics of the chemo-mechanical cycle, to the prevailing mechanical conditions. Intriguingly at higher loads the main power stroke associated with the release of phosphate (or possibly an isomerization step of an ADP bound state following the release of phosphate) was reversed and the lever arm of the motor was moving back and forth between pre-power stroke and post-power stroke conformations (Sellers and Veigel, 2010). These experiments were a direct demonstration of a “rocking cross bridge” as proposed by Huxley and Simmons (1971) for the mechanics of muscle. For myosin V it provided a molecular explanation for the change in directionality of the processive motor at forces equal to or larger than the stall force. At even higher loads the myosin V heads detached from actin, irrespective of them being in a pre or post power stroke conformation.

The question of whether there is a tight coupling between cross bridge performance and ATP utilization was addressed in several studies. Ishijima et al. used optical trapping combined with fluorescence detection of cy3-ATP and came to the conclusion that skeletal muscle myosin II S1 might not have a tight one-to-one coupling between the mechanical and chemical cycles (Ishijima et al., 1998). Sakamoto et al. simultaneously observed the processive movement of myosin V along with the binding and dissociation of another fluorescent ATP analog, deac-aminoATP (Sakamoto et al., 2008). They found a tight one-to-one coupling of nucleotide utilization for each step in the single molecule fluorescence assay and found that the leading head had little tendency to release nucleotide as long as the trail head was attached. This is consistent with load dependence effectively gating the kinetics of the two heads to aid in the processive movement, as has been suggested in the earlier single molecule mechanical experiments (Veigel et al., 2001).

## Future areas of interest

There are many outstanding questions still remaining to fully understand muscle fiber mechanics. How do disease states alter cross-bridge mechanics? What are the rate constants for cross-bridges in isometrically contracting skeletal muscle fibers? How do the rate constants in the isotonically contracting muscle change and by how much as shortening velocity is altered? How do the rate constants in eccentrically contracting muscle change with rate of stretch and to what extent are the various populations of attached cross bridges altered? How are rate constants in contracting muscle altered by the extent of calcium activation or is the only change a rate in which the cross-bridges attach to exposed actin filaments? Finally a broad question, to what extent is skeletal muscles contractility altered by changes in diet, hormones, exercise, age, use, and innervations? It is clear how reductionist methods can address some of these questions. Several labs are using biophysical methods to probe myosins bearing mutations corresponding to ones found in diseased hearts (Palmiter et al., 2000; Sommese et al., 2013) and it is possible to probe actin filaments with bound troponin-tropomyosin in the optical trap (Kad et al., 2005). Some of the other questions require precise control of the force experience by the myosin, but there is interesting work demonstrating the feasibility of this approach (Takagi et al., 2006; Capitanio et al., 2012).

## Summary

To understand how muscle works the vertebrate skeletal muscle systems has many advantages. However, the inability to make mutations within the myosin molecule in muscle, coupled with the fast and low duty ratio kinetics has made it challenging to understand the system at a molecular level. Using a comparative approach with recombinantly expressed non-muscle myosins, combined with a host of biochemical and biophysical techniques applied to purified myosin has allowed researchers to unravel how muscle works at a molecular level.

### Conflict of interest statement

The authors declare that the research was conducted in the absence of any commercial or financial relationships that could be construed as a potential conflict of interest.
